# Brain metastases from hepatopancreatobiliary malignancies

**DOI:** 10.1007/s10585-023-10201-1

**Published:** 2023-03-22

**Authors:** Elisabeth S. Bergen, Alexander Friedrich, Peter Scherleitner, Pedro Ferreira, Barbara Kiesel, Georg Widhalm, Barbara Kiesewetter, Franziska Eckert, Gerald W. Prager, Matthias Preusser, Anna S. Berghoff

**Affiliations:** 1 Division of Oncology, Department of Medicine I, Medical University of Vienna, Waehringer Guertel 18-20, 1090 Vienna, Austria; 2 Department of Neurosurgery, Medical University of Vienna, Vienna, Austria; 3 Department of Radiation Oncology, Medical University of Vienna, Vienna, Austria; 4 Christian Doppler Laboratory for Personalized Immunotherapy, Department of Medicine I, Medical University of Vienna, Vienna, Austria

**Keywords:** Brain metastases, Gastrointestinal tumors, Hepatopancreatobiliary cancer

## Abstract

While colorectal and gastroesophageal cancer represent the two gastrointestinal (GI) tumor entities with the highest incidence of brain metastatic (BM) disease, data on the clinical course of BM patients from hepatopancreatobiliary malignancies are rare. Patients with cholangiocarcinoma (CCA), hepatocellular carcinoma (HCC), pancreatic ductal adenocarcinoma (PDAC) and gastroenteropancreatic neuroendocrine neoplasms (GEP NEN). Treated for BM between 1991 and 2017 at an academic care center were included. Brain metastases-free survival (BMFS) was defined as interval from first diagnosis until BM development. Overall survival (OS) was defined as interval from diagnosis of BM until death or last date of follow-up. Outcome was correlated with clinical and treatment factors. 29 patients from overall 6102 patients (0.6%) included in the Vienna Brain Metastasis Registry presented with BM from hepatopancreatobiliary primaries including 9 (31.0%) with CCA, 10 (34.5%) with HCC, 7 (24.1%) with PDAC and 3 (10.3%) with GEP NEN as primary tumor. Median BMFS was 21, 12, 14 and 7 months and median OS 4, 4, 6 and 4 months, respectively. Karnofsky Performance Status (KPS) below 80% (*p* = *0.08*), age above 60 years (*p* = *0.10*) and leptomeningeal carcinomatosis (LC) (*p* = *0.09*) diagnosed concomitant to solid BM showed an inverse association with median OS (Cox proportional hazards model). In this cohort of patients with BM from hepatopancreatobiliary tumor entities, prognosis was shown to be very limited. Performance status, age and diagnosis of LC were identified as negative prognostic factors.

## Background

The incidence of brain metastatic (BM) disease is increasing among several tumor entities potentially due to better diagnostic modalities and an improved control of extracranial disease achieved by systemic therapies [1, 2]. Lung cancer, breast cancer and melanoma patients thereby exhibit the highest incidence of BM with up to 50%, 15% and 10%, respectively and therefore available data on the clinical course of disease of these patients in the meanwhile seem well explored [1, 3, 4]. The prognosis of patients with BM varies extensively not only between different tumor entities, but even between distinct tumor subtypes. Age, performance status, presence or absence of extracranial metastases as well as number of BM were shown to represent the most important clinical prognosticators of survival in these patients as described by the Graded Prognostic Assessment (GPA) score [5].

Since patients with gastrointestinal (GI) primary tumors represent approximately only 6% of BM patients, clinical data is very limited. Patients with colorectal and gastroesophageal tumors are most likely to develop BM lesions throughout their course of disease [6]. Once BM are diagnosed in these patients, overall survival (OS) remains very limited with a median of 8 months among all GI primaries. Here, the same clinical biomarkers as in breast, lung and melanoma patients were recently identified as independent prognosticators for OS, which has been summarized within the graded prognostic assessment for gastrointestinal cancers (GI-GPA) [7]. However, other primary GI tumors beside colorectal and gastroesophageal cancer have been barely investigated and therefore data on these patients remain scarce after diagnosis of BM.

Within the present study we therefore aimed to describe the clinical characteristics as well as the outcome of patients with BM from rare GI cancer treated at our tertiary care center. We took advantage of the joined focus on GI cancers as well as the Vienna Brain Metastasis Registry to identify patients with GI cancers less frequently developing BM compared to colorectal and gastroesophageal tumors. We investigated clinical risk factors and different BM therapies associated with OS. We thereby focused on patients with cholangio- (CCA), hepatocellular (HCC) and pancreatic ductal adenocarcinoma (PDAC) as well as gastroenteropancreatic neuroendocrine neoplasms (GEP NEN).

## Methods

### Patients

**Fig. 1:**
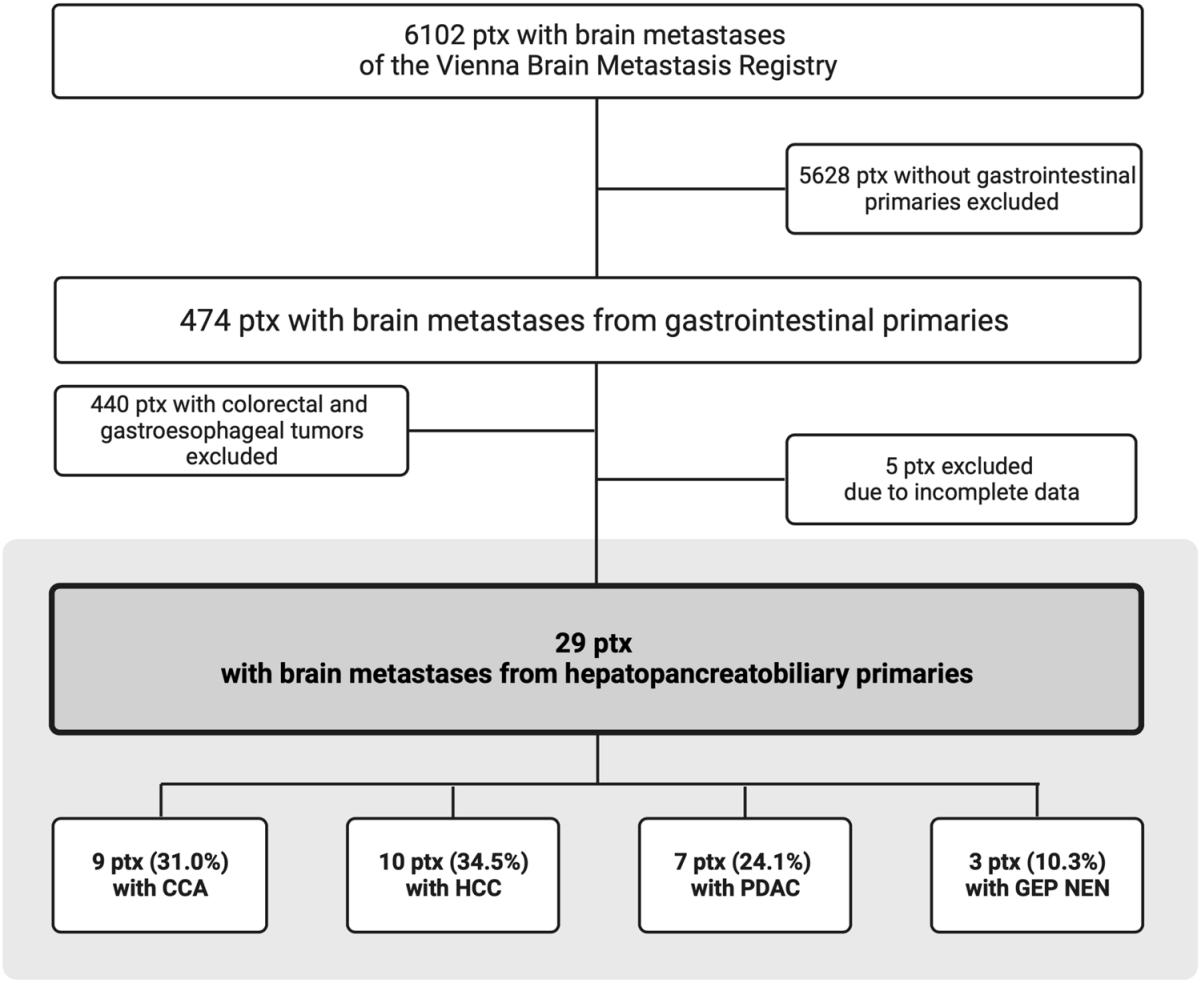
Flowchart (*CCA* cholangiocarcinoma, *GEP NEN* gastroenteropancreatic neuroendocrine neoplasm, *HCC* hepatocellular carcinoma, *PDAC* pancreatic ductal adenocarcinoma, *ptx* patients)

From 6102 patients with BM registered in the Vienna Brain Metastasis Registry, 34 patients (0.6%) had a CCA, HCC, PDAC or GEP NEN as primary tumor. After exclusion of patients with missing clinical data, 29 patients remained for the final analysis of this study (Fig. 1). All of these patients were treated between 1991 and 2017 at the Medical University of Vienna. If leptomeningeal carcinomatosis (LC) was present concomitantly to diagnosis of parenchymal BM, patients were also eligible for inclusion. Information relating to patient demographics, case history, and survival was collected by retrospective chart review. This study was conducted in accordance with the Declaration of Helsinki and approval by the institutional review board (IRB) was obtained (1167/2019).

All patients were managed by a dedicated team of GI cancer and BM specialists. Treatment was performed according to best clinical evidence and according to current standard of care at the respective time.

### Statistical analysis

The primary aim of this study was to describe the clinical characteristics of a cohort of hepatopancreatobiliary cancer patients (CCA, HCC, PDAC, GEP NEN) with BM in a descriptive manner. The secondary aim was to identify prognostic clinical factors with regards to patient’s outcome after diagnosis of BM as well as to compare different BM therapies. Brain metastases-free survival (BMFS) was defined as the interval from diagnosis of GI cancer until diagnosis of BM. OS was defined as interval from first diagnosis of BM until death or last date of follow-up. Both endpoints were estimated with the Kaplan–Meier product limit method. To test for differences between survival curves, the log-rank test was used. Two-tailed *p* values < 0.05 were considered to indicate statistical significance.

The recently updated GI-GPA including KPS (< 80, 80, 90–100), age (< 60, ≥ 60 years), extracranial metastases (present, absent) and number of BM (1, 2–3, > 3) represents the so far best established prognosticator of outcome in GI BM patients [7]. Therefore, we predefined a priori the inclusion of these 4 clinical parameters together with the GI-GPA as well as other important clinical parameters (gender, visceral metastases, tumor entity, status of extracranial disease at diagnosis of BM, symptomatic BM, LC) into the multivariate model, depending on their significance in the univariate analysis. A multivariate analysis was performed using the Cox Regression model. Due to the exploratory and hypothesis-generating design of the present study, no adjustment for multiple testing was applied and no formal sample-size calculation was conducted [8]. All statistics were calculated using statistical package for the social sciences (SPSS^®^) 28.0 software (SPSS Inc., Chicago, IL, USA).

## Results

Among 6102 patients with BM from the Vienna Brain Metastasis Registry 474 (7.8%) presented with a GI primary tumor. 440 patients with colorectal or gastroesophageal primaries and 5 patients with incomplete data were excluded. Therefore, 29 patients were available for the present study comprising 9 patients with CCA (31.0%), 10 patients with HCC (34.5%), 7 patients with PDAC (24.1%) and 3 patients with GEP NEN (10.3%) as primary tumor diagnosis (Fig. 1). BM were diagnosed in 4/29 patients (13.8%) before the year 2000, in 7/29 patients (24.1%) between 2000 and 2010 and in 18/29 patients (62.1%) after the year 2010. Median BMFS was 18 months (range 0 to 111) among all included patients. Median OS from first diagnosis was 21 months (range 1 to 115), from diagnosis of metastatic disease 16 months (range 0 to 71) and from diagnosis of BM 4 months (range 0 to 29).

### CCA patients

**Fig. 2:**
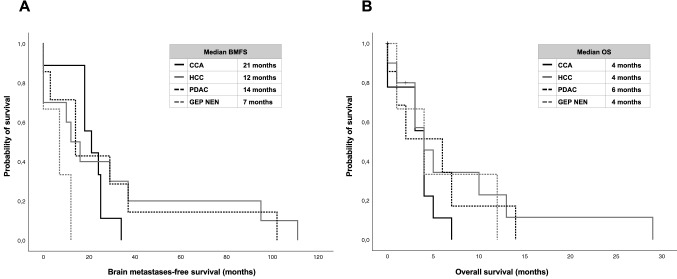
Kaplan–Meier curves of **A** brain metastasis-free survival (BMFS) and **B** overall survival (OS) according to different tumor entities (*BMFS* brain metastasis-free survival, *CCA* cholangiocarcinoma, *GEP NEN* gastroenteropancreatic neuroendocrine neoplasm, *HCC* hepatocellular carcinoma, *OS* overall survival, *PDAC* pancreatic ductal adenocarcinoma)

Median age of CCA patients at diagnosis of BM was 69 years (range 52 to 76) and median KPS 70% (range 40 to 80). Patients had a median number of 1 extracranial metastatic site (range 0 to 3) at diagnosis of BM and were treated with a median number of 1 line of systemic therapy (range 0 to 3) before. Before diagnosis of BM, liver metastases were present in 4/9 patients (44.4%), lung metastases in 4/9 patients (44.4%) and peritoneal metastases in 1/9 patients (11.1.%). At diagnosis of BM, extracranial disease was progressive in 5/9 patients (55.6%), stable in 1/9 patients (11.1%) and in remission in 3/9 patients (33.3%). All 9 patients (100.0%) were symptomatic with regards to BM diagnosis. Median number of BM lesions was 1 (range 1 to 4). 2/9 patients (22.2%) presented with LC concomitant to solid BM diagnosis. CCA patient characteristics are listed within Table 1. Median BMFS was 21 months (range 0 to 34) and median OS 4 months (range 0 to 7) (Fig. 2).

**Table 1:** Patient’s characteristics according to different tumor entities

	Total	Tumor entity			
		CCA	HCC	PDAC	GEP NEN
	n = 29	n = 9	n = 10	n = 7	n = 3
Gender					
Male	15 (51.7%)	5 (55.6%)	8 (80.0%)	1 (14.3%)	1 (33.3%)
Female	14 (48.3%)	4 (44.4%)	2 (20.0%)	6 (85.7%)	2 (66.7%)
Metachronous/synchronous metastases					
Metachronous	18 (62.1%)	6 (66.7%)	6 (60.0%)	6 (85.7%)	0 (0.0%)
Synchronous	11 (37.9%)	3 (33.3%)	4 (40.0%)	1 (14.3%)	3 (100.0%)
Nb of systemic therapy lines before diagnosis of BM					
Median	1	1	0	1	1
Range	0–6	0–3	0–1	0–6	0–2
Time period of BM diagnosis (years)					
< 2000	4 (13.8%)	2 (22.2%)	1 (10.0%)	0 (0.0%)	1 (33.3%)
2000–2010	7 (24.1%)	1 (11.1%)	4 (40.0%)	2 (28.6%)	0 (0.0%)
> 2010	18 (62.1%)	6 (66.7%)	5 (50.0%)	5 (71.4%)	2 (66.7%)
Age at diagnosis of BM (years)					
Median	65	69	61	64	70
Range	44–82	52–76	48–81	44–82	45–73
KPS at diagnosis of BM					
Median	80	70	80	80	70
Range	40–90	40–80	50–90	60–80	70–80
Status of extracranial disease at diagnosis of BM					
Remission	7 (25.9%)	3 (33.3%)	2 (22.2%)	1 (16.7%)	1 (33.3%)
Stable disease	5 (18.5%)	1 (11.1%)	3 (33.3%)	1 (16.7%)	0 (0.0%)
Progressive disease	15 (55.6%)	5 (55.6%)	4 (44.4%)	4 (66.7%)	2 (66.7%)
Missing	2	0	1	1	0
Nb of metastatic sites at diagnosis of BM					
Median	1	1	0	1	3
Range	0–3	0–3	0–3	0–3	0–3
Nb of BM					
1	14 (48.3%)	5 (55.6%)	7 (70.0%)	1 (14.3%)	1 (33.3%)
2–3	5 (17.2%)	0 (0.0%)	1 (10.0%)	3 (42.9%)	1 (33.3%)
> 3	10 (34.5%)	4 (44.4%)	2 (20.0%)	3 (42.9%)	1 (33.3%)
Symptomatic BM					
Yes	23 (79.3%)	9 (100.0%)	6 (60.0%)	7 (100.0%)	1 (33.3%)
No	6 (20.7%)	0 (0.0%)	4 (40.0%)	0 (0.0%)	2 (66.7%)
Leptomeningeal carcinomatosis at diagnosis of BM					
Yes	4 (13.8%)	2 (22.2%)	1 (10.0%)	1 (14.3%)	0 (0.0%)
No	25 (86.2%)	7 (77.8%)	9 (90.0%)	6 (85.7%)	3 (100.0%)
Initial therapy of BM					
Gamma knife	11 (37.9%)	3 (33.3%)	4 (40.0%)	3 (42.9%)	1 (33.3%)
Surgery + RTX	5 (17.2%)	1 (11.1%)	3 (30.0%)	0 (0.0%)	0 (0.0%)
Surgery without RTX	2 (6.9%)	0 (0.0%)	1 (10.0%)	1 (14.3%)	1 (33.3%)
WBRT	6 (20.7%)	4 (44.4%)	1 (10.0%)	0 (0.0%)	1 (33.3%)
BSC	5 (17.2%)	1 (11.1%)	1 (10.0%)	3 (42.9%)	0 (0.0%)
Intracranial PD after initial therapy for BM					
Yes	6 (20.7%)	1 (11.1%)	2 (20.0%)	3 (42.9%)	0 (0.0%)
No	23 (79.3%)	8 (88.9%)	8 (80.0%)	4 (57.1%)	3 (100.0%)
Systemic therapy lines after diagnosis of BM					
Median	0	0	0	0	1
Range	0–2	0–2	0–2	0–2	0–1
Reason of death*					
BM	6 (35.3%)	3 (42.9%)	2 (50.0%)	1 (20.0%)	0 (0.0%)
Extracranial disease	9 (47.1%)	4 (57.1%)	1 (25.0%)	2 (40.0%)	2 (100.0%)
Both	3 (17.6%)	0 (0.0%)	1 (25.0%)	2 (40.0%)	0 (0.0%)
Missing	11	2	6	2	1

*BM* brain metastases, *BSC* best supportive care, *CCA* cholangiocarcinoma, *GEP NEN* gastroenteropancreatic neuroendocrine neoplasm, *HCC* hepatocellular carcinoma, *KPS* Karnofsky Performance Status, *nb* number, *PD* progressive disease, *PDAC* pancreatic ductal adenocarcinoma, *WBRT* whole brain radiotherapy

*As defined by progressive disease in a restaging within 6 weeks before death

### HCC patients

Median age of HCC patients at diagnosis of BM was 61 years (range 48 to 81) and median KPS 80% (range 50 to 90). Patients had a median number of 0 extracranial metastatic sites (range 0 to 3) at diagnosis of BM and were treated with a median number of 0 lines of systemic therapy (range 0 to 3) before. Before diagnosis of BM, liver metastases were present in 3/10 patients (30.0%), lung metastases in 3/10 patients (30.0%) and peritoneal metastases in 0/10 patients (0.0%). At diagnosis of BM, extracranial disease was progressive in 4/10 patients (44.4%), stable in 3/10 patients (33.3%) and in remission in 2/10 patients (22.2%) (*1 unknown*). Six/10 patients (60.0%) were symptomatic and 4/10 patients (40.0%) asymptomatic with regards to BM diagnosis. Median number of BM lesions was 1 (range 1 to 4). One/10 patients (10.0%) presented with LC concomitant to solid BM diagnosis. HCC patient characteristics are listed within Table 1. Median BMFS was 12 months (range 0 to 111) and median OS 4 months (range 0 to 29) (Fig. 2).

### PDAC patients

Median age of PDAC patients at diagnosis of BM was 64 years (range 44 to 82) and median KPS 80% (range 60 to 90). Patients had a median number of 1 extracranial metastatic site (range 0 to 3) at diagnosis of BM and were treated with a median number of 1 line of systemic therapy (range 0 to 6) before. Before diagnosis of BM, liver metastases were present in 1/7 patients (16.7%, 1 missing), lung metastases in 2/7 patients (33.3, 1 missing) and peritoneal metastases in 0/7 patients (0.0%, 1 missing). At diagnosis of BM, extracranial disease was progressive in 4/7 patients (66.7%), stable in 1/7 patients (16.7%) and in remission in 1/7 patients (16.7%) (*1 unknown*). All of the 7 patients (100.0%) were symptomatic with regards to BM diagnosis. Median number of BM lesions was 3 (range 1 to 10). One/7 patients (14.3%) presented with LC concomitant to solid BM diagnosis. PDAC patient characteristics are listed within Table 1. Median BMFS was 14 months (range 0 to 102) and median OS 6 months (range 0 to 14) (Fig. 2).

### GEP NEN patients

Among the 3 GEP NEN patients, one patient presented with a neuroendocrine tumor of the pancreas, one patient with a neuroendocrine carcinoma of the pancreas and one patient with a neuroendocrine carcinoma of the small intestine. Median age of GEP NEN patients at diagnosis of BM was 70 years (range 45 to 73) and median KPS 70% (range 70 to 80). Patients had a median number of 3 extracranial metastatic sites (range 0 to 3) at diagnosis of BM and were treated with a median number of 1 line of systemic therapy (range 0 to 2) before. Before diagnosis of BM, liver metastases were present in 2/3 patients (66.7%), lung metastases in 1/3 patients (33.3%) and peritoneal metastases in 0/3 patients (0.0%). At diagnosis of BM, extracranial disease was progressive in 2/3 patients (66.7%) and in remission in 1/13 patients (33.3%). One/3 patients (33.3%) was symptomatic and 2/3 patients (66.7%) asymptomatic with regards to BM diagnosis. Median number of BM lesions was 3 (range 1 to 4). None/3 patients (0.0%) presented with LC concomitant to solid BM diagnosis. GEP NEN patient characteristics are listed within Table 1. Median BMFS was 7 months (range 0 to 12) and median OS 4 months (range 1 to 12) (Fig. 2).

### BM treatment in patients with BM from rare GI tumors

**Fig. 3:**
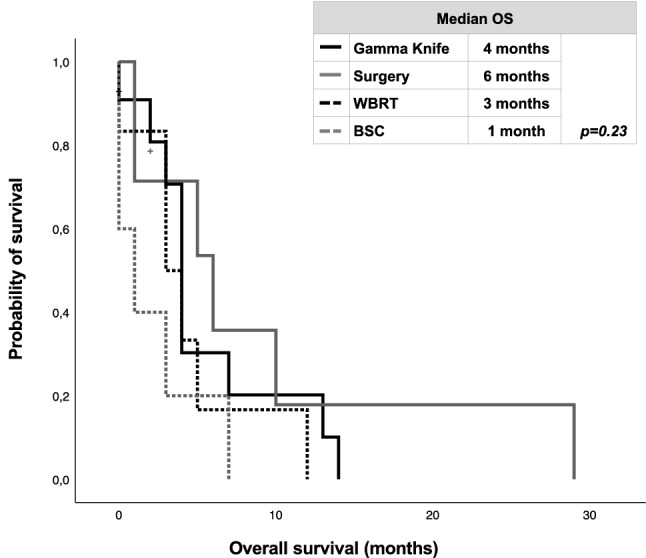
Kaplan–Meier curves of overall survival (OS) according to treatment of brain metastases (*BSC* best supportive care, *OS* overall survival, *WBRT* whole brain radiotherapy)

In the overall patient’s cohort, 11/29 (37.9%) of patients were treated with stereotactic radiosurgery (SRS) as initial therapy for BM, 7 (24.1%) with neurosurgical resection, 6 (20.7%) with whole brain radiotherapy (WBRT) and 5 (17.2%) with best supportive care (BSC). Median OS after BM was 4 months in patients treated with GK, 6 months with neurosurgical resection, 3 months with WBRT and 1 month with BSC (*p* = 0.23; log-rank test) (Fig. 3)*.*

### Identification of prognostic biomarkers

Uni- and multivariable analyses of the overall patient cohort were performed to identify prognosticators of OS. Here, a KPS under 80% (*p* = *0.08*), an age above 60 years (*p* = 0.10) and LC (*p* = 0.09) at diagnosis of BM were identified as being significantly associated with OS according to univariable analysis. No significant association was observed with regards to number of BM lesions or extracranial metastases (*p* > 0.1). Within multivariable analysis none of the factors remained independently and significantly associated with OS. Results of uni- and multivariable analyses are listed within Table 2.

**Table 2:** Effect on overall survival (OS) according to different clinical factors

	Overall patient cohort			
	Univariable analysis		Multivariable analysis	
	HR (95% CI)	p-value	HR (95% CI)	p-value
Gender				
Female	1.25 (0.58–2.71)	*0.57*		
Male	1			
KPS at BM diagnosis				
< 80	4.19 (0.84–20.86)	***0.08***	4.16 (0.82–20.98)	*0.09*
80	1.76 (0.39–8.02)	*0.46*	1.52 (0.32–7.15)	*0.60*
90–100	1		1	
Age at BM diagnosis				
< 60 years	1		1	
≥ 60 years	2.09 (0.85–5.12)	***0.10***	1.92 (0.78–4.71)	*0.15*
Extracranial metastases at diagnosis of BM				
Absent	1			
Present	1.27 (0.55–2.91)	*0.58*		
Visceral metastases before diagnosis of BM				
Yes	1.33 (0.59–2.98)	*0.49*		
No	1			
Tumor entity				
CCA	1			
HCC	0.52 (0.19–1.42)	*0.20*		
PDAC	0.64 (0.22–1.91)	*0.43*		
GEP NEN	0.67 (0.18–2.58)	*0.56*		
Number of BM lesions				
1	1			
2–3	1.20 (0.38–3.81)	*0.76*		
> 3	2.01 (0.85–4.76)	*0.11*		
GI-GPA				
Class 1	1			
Class 2	1.44 (0.27–7.63)	*0.67*		
Class 3	2.14 (0.42–11.04)	*0.36*		
Class 4	2.93 (0.63–13.68)	*0.17*		
Status of extracranial disease at diagnosis of BM				
Remission	1			
Stable	0.82 (0.23–2.96)	*0.76*		
Progressive	2.12 (0.75–5.96)	*0.16*		
Symptomatic BM				
Yes	1.12 (0.44–2.84)	*0.81*		
No	1			
Leptomeningeal carcinomatosis at diagnosis of BM				
Yes	2.67 (0.86–8.26)	***0.09***	3.18 (0.97–10.46)	*0.06*
No	1		1	

Univariable and multivariable analysis stratified by study cox proportional hazard models

*BM* brain metastases, *CCA* cholangiocarcinoma, *CI* confidence interval, *GEP NEN* gastroenteropancreatic neuroendocrine neoplasm, *GI GPA* graded prognostic assessment for gastrointestinal cancers, *HCC* hepatocellular carcinoma, *HR* hazard ratio, *KPS* Karnofsky Performance Status, *PDAC* pancreatic ductal adenocarcinoma

## Discussion

Within the present study we aimed to characterize patients with BM from hepatopancreatobiliary tumor entities. Among over 6000 BM patients registered in the Vienna Brain Metastasis Registry representing one of the largest datasets on this distinct patient population worldwide, only 29 patients had a rare GI tumor as primary disease. As this represents such an infrequent cohort of patients, our data may provide insight into the clinical course of disease and support treatment decisions in daily clinical practice.

Only recently, the GI-GPA postulated the KPS, age, extracranial disease and number of BM lesions as major prognostic factors after diagnosis of BM in patients with GI primaries [7]. Prognostic biomarkers for survival after diagnosis of BM identified in our study were the KPS, age and the presence of LC, whereas presence or absence of extracranial disease as well as number of BM lesions were not associated with survival and thus seem less relevant in the present cohort. A controlled extracranial disease, however, was shown to act as highly important prognosticator among several tumor entities even after BM diagnosis [9]. Patients with a progressive extracranial disease in our study yielded also an—albeit not significantly—worse survival compared to patients in remission or stable disease within the multivariable analysis (HR 2.12). Therefore, the control of extracranial metastases may be prognostically more relevant after diagnosis of BM than its absence or presence. Another explanation why the extracranial disease may be less relevant in the present patient cohort once BM are diagnosed may be the limited prognosis thereafter.

Interestingly, we observed a rising incidence of BM over the last decades. This is well in line with larger clinical trials including different tumor entities pointing into the same direction [10]. Several factors may contribute to the rising number of BM. There certainly has been a significant improvement of diagnostic imaging techniques. Moreover, awareness of treating physicians to perform cranial imaging in case of BM related symptoms even in tumor entities rarely developing BM may have increased as well. As larger clinical trials observed that especially the number of asymptomatic BM patients is rising, screening implementation may also be important in tumor entities with a high BM probability. Most importantly, prognosis of BM patients improved remarkably over the last decades most likely due to an improvement in local therapies as well as a broader spectrum of systemic therapies available [11].

Compared to other tumor entities, however, the OS after BM of 4 months observed in our study was considerably lower. Patients with BM exhibit the most favorable prognosis with breast, lung and renal cancer primaries with a median of 16, 15 and 12 months, respectively [12]. But also in the cohort of patients included for the GI-GPA assessment, the observed median OS was 8 months [7]. Since included patients consisted primarily of gastroesophageal and colorectal cancer patients, outcome with CCA, HCC, PDAC and GEP NEN may therefore even be worse. One reason for the favorable prognosis of BM patients with other cancer entities most likely lies in an efficient systemic disease control achieved by applied systemic therapies. Furthermore, there is increasing evidence about remarkable intracranial responses achieved by several targeted therapies in certain tumor entities [13–16]. For patients with CCA, PDAC and GEP NEN, however, targeted therapy approaches so far have not entered clinical routine and intracranial efficacy of chemotherapeutic agents remains limited. Patients with HCC included into the present study were treated before 2017 and thus immunotherapy and the vascular endothelial growth factor receptor (VEGFR) targeted antibody bevacizumab, potentially having some intracranial activity, not yet used. Another reason for the limited prognosis of patients included into the present study may be the generally more aggressive nature of their tumor entities and therefore intracranial tumor cells may bare a more aggressive tumor biology as well.

Patients treated with neurosurgical resection in our study had the best prognosis after BM followed by STS, WBRT and BSC. This is well in line with actual treatment guidelines in this field, that targeted local therapies like resection and STS should be the preferred treatment approach in patients with oligometastatic disease whenever technically feasible [1]. Most operated patients included into this study were diagnosed and treated between the year 2000 and 2010, and postoperative radiation was not standard of care to that time. Therefore, intracranial tumor control may be even favorable nowadays with STS after resection, even though survival times may not be longer compared to observation alone according to a randomized clinical trial [17]. WBRT had been the first-line treatment of choice in patients with multiple BM historically. However, as observed within this study and also within previous studies including other cancer primaries, its efficacy seems comparable to BSC [18, 19]. Therefore, and due to its unfavorable toxicity profile, systemic therapies became the preferred treatment option whenever available in this indication in patients with multiple, asymptomatic BM lesions. In patients with GI cancer entities, however, WBRT may still be considered especially in patients with multiple BM.

Our study clearly faces some limitations manly due to its retrospective design. Moreover, sample sizes of patients within the included tumor subgroups were low and therefore the detected results have to be interpreted with caution. Also, the inclusion period of patients comprised 26 years and therefore respective standards of diagnostic and treatment modalities may be heterogenous. Nevertheless, patients had the benefit of being treated in a GI specialized tertiary care center from their initial diagnosis onwards during their complete course of disease and beyond their BM diagnosis. This gains valuable insight in this rare event to put the prognosis of patients into context. Moreover, this study represents the first characterization of patients with hepatopancreatobiliary cancer entities after diagnosis of BM.

In conclusion, patients with CCA, HCC, PDAC and GEP NEN still face a rather limited OS prognosis of 4 months after diagnosis of BM. Established prognostic factors were shown to apply also for these distinct patient populations. Performance status, age and concomitant LC thereby were the most important clinical factors. Overall, patients with targeted local therapies for BM exhibited the best outcome.

## Abbreviations

BM: Brain metastases
BMFS: Brain metastases-free survival
BSC: Best supportive care
CCA: Cholangiocarcinoma
GEP NEN: Gastroenteropancreatic neuroendocrine neoplasms
GI: Gastrointestinal
GI-GPA: Graded prognostic assessment for gastrointestinal cancers
GPA: Graded Prognostic Assessment
HCC: Hepatocellular carcinoma
IRB: Institutional review board
KPS: Karnofsky Performance Status
LC: Leptomeningeal carcinomatosis
OS: Overall survival
PDAC: Pancreatic ductal adenocarcinoma
SPSS: Statistical package for the social sciences
SRS: Stereotactic radiosurgery
VEGFR: Vascular endothelial growth factor receptor
WBRT: Whole brain radiotherapy
